# Whole-Genome Sequencing of *Salmonella enterica* subsp. *enterica* Serovar Ouakam Isolated from Ground Turkey

**DOI:** 10.1128/genomeA.01618-15

**Published:** 2016-01-21

**Authors:** Daya Marasini, Usama H. Abo-Shama, Mohamed K. Fakhr

**Affiliations:** a Department of Biological Science, The University of Tulsa, Tulsa, Oklahoma, USA; b Microbiology and Immunology Department, Faculty of Veterinary Medicine, Sohag University, Sohag, Egypt

## Abstract

In this report, we announce the first whole-genome sequencing of *Salmonella enterica* subsp. *enterica* serovar Ouakam strain GNT-01, isolated from ground turkey retail meat. The strain has a chromosome of 5,088,451 bp long, with a G+C content of 52.3%, and a plasmid of 109,715 bp.

## GENOME ANNOUNCEMENT

*Salmonella* is one of the leading causes of foodborne illness, causing approximately 1.2 million illnesses in the United States, with 380 deaths and nearly 8,000 reported infections yearly (1, 2). Nontyphoidal *Salmonella* spp. are the leading cause of hospitalization among organisms causing foodborne illnesses, being responsible for 35% of admission cases (3). *Salmonella enterica* subsp. *enterica* serovar Ouakam is one of the serotypes associated with foodborne illnesses. *Salmonella* Ouakam was isolated from food samples in Morocco (4) and from humans in Africa (5). It was also isolated from swine and their environment (6) and chicken farms (7). *Salmonella* Ouakam was also reported in humans and feed mills in Sweden (8).

To our knowledge, there are no whole-genome sequences of *S. enterica* serovar Ouakam in GenBank. In this report, we announce the first whole-genome sequence of *S. enterica* subsp. *enterica* serovar Ouakam strain GNT-01, isolated from retail ground turkey meat.

The genomic DNA was isolated from an overnight culture of tryptic soya broth using the Qiagen DNeasy Blood and tissue kit (Qiagen, Inc., Valencia, CA). The sample library was prepared using the Nextera XT sample preparation kit and sequenced using the MiSeq desktop sequencer (Illumina, Inc., San Diego, CA). The sequences were assembled using CLC Genomics Workbench, and the contigs were arranged using the Microbial Genome Finishing Module from that program (Qiagen, Inc.). The annotation was first performed using Rapid Annotations using Subsystems Technology (RAST) and then by the NCBI Prokaryotic Genome Annotation Pipeline.

The main chromosome was found to be 5,088,451 bp long, with a G+C content of 52.3%. In addition to the chromosome, a circular large plasmid of 109,715 bp in size, named pDM01, was found in the strain. The annotation performed by NCBI Prokaryotic Genome Annotation Pipeline revealed the presence of a total of 5,033 genes with 4,713 coding sequences (CDSs), of which 115 coding sequences were present on the plasmid. There were a total of 115 RNA-coding genes, 205 pseudogenes, and 128 frameshifted genes. The plasmid was found to contain aminoglycoside resistance genes. The highest similarity of the chromosomal sequence was seen with *S. enterica* serovar Newport strain CVM 21550 (accession no. CP010283.1), with 99% nucleotide identity at 95% coverage. The plasmid sequence shared the highest similarity with *S. enterica* serovar Heidelberg plasmid pSH 1148_107 (accession no. JN983049.1), with 99% homology for 94% coverage. The size of the plasmid is slightly larger than that reported in *Salmonella* Heidelberg, with minimal variation in the sequence. To our knowledge, this is the first whole-genome sequence of *S. enterica* subsp. *enterica* serovar Ouakam to be deposited in GenBank.

### Nucleotide sequence accession numbers.

The chromosomal and the plasmid sequences were deposited in the GenBank under accession numbers CP012038 and CP012039, respectively.
